# Tilting the balance toward motor symptoms: Determinants of caregiver burden in late-stage Parkinson’s disease

**DOI:** 10.1016/j.ibneur.2026.05.003

**Published:** 2026-05-15

**Authors:** Laura Culicetto, Gabriele Triolo, Roberta Lombardo, Daniela Ivaldi, Lilla Bonanno, Fabio Mauro Giambò, Giuseppe Di Lorenzo, Chiara Sorbera, Angelo Quartarone, Silvia Marino, Viviana Lo Buono

**Affiliations:** a IRCCS Centro Neurolesi Bonino Pulejo, Italy; b University of Messina, Italy

**Keywords:** Parkinson, Late-stage Disease, Caregiver Burden, Motor Symptoms, Non-motor, Symptoms, Autonomy

## Abstract

**Background:**

Caregiver burden in Parkinson’s disease (PD) is influenced by both motor and non-motor symptoms; however, the relative contribution of these domains in late-stage PD remains unclear. This study aimed to examine the clinical determinants of caregiver burden and to explore whether motor-functional impairment plays a relevant role in advanced PD.

**Methods:**

Thirty patient–caregiver dyads were enrolled. Individuals with late-stage PD (Hoehn and Yahr stage IV) underwent a comprehensive clinical evaluation assessing motor performance, cognitive functioning and functional autonomy. Caregiver burden was assessed using a multidimensional self-report questionnaire. Associations were analyzed using Spearman correlations with false discovery rate correction. Multivariable linear regression models were fitted to explore independent contributors across motor, sleep, and QoL domains.

**Results:**

Caregiver burden was associated with longer caregiving duration (ρ = 0.81, p_FDR < 0.001), reduced functional autonomy (IADL: ρ = −0.76; ADL: ρ = −0.63; both p_FDR < 0.001), and poorer motor performance (ρ = −0.67, p_FDR < 0.001). Among non-motor domains, burden correlated with worse mobility-related QoL (ρ = 0.68, p_FDR < 0.001) and sleep disturbance (ρ = 0.57, p_FDR = 0.003), whereas cognition and affective symptoms were not significantly associated after correction. The motor-focused regression model showed slightly higher explanatory power (R² = 0.80).

**Conclusions:**

In advanced PD, caregiver burden is driven by motor-functional dependence and loss of autonomy, with sleep disturbance and perceived mobility-related impairment further contributing. These findings underscore the role of motor-functional disability in shaping caregiver strain in late-stage disease, highlighting the need for integrated, caregiver-centered management strategies.

**Background:**

Caregiver burden in Parkinson’s disease (PD) is influenced by both motor and non-motor symptoms; however, the relative contribution of these domains in late-stage PD remains unclear. This study aimed to examine the clinical determinants of caregiver burden and to explore whether motor-functional impairment plays a relevant role in advanced PD.

**Methods:**

Thirty patient–caregiver dyads were enrolled. Individuals with late-stage PD (Hoehn and Yahr stage IV) underwent a comprehensive clinical evaluation assessing motor performance, cognitive functioning and functional autonomy. Caregiver burden was assessed using a multidimensional self-report questionnaire. Associations were analyzed using Spearman correlations with false discovery rate correction. Multivariable linear regression models were fitted to explore independent contributors across motor, sleep, and QoL domains.

**Results:**

Caregiver burden was associated with longer caregiving duration (ρ = 0.81, p_FDR < 0.001), reduced functional autonomy (IADL: ρ = −0.76; ADL: ρ = −0.63; both p_FDR < 0.001), and poorer motor performance (ρ = −0.67, p_FDR < 0.001). Among non-motor domains, burden correlated with worse mobility-related QoL (ρ = 0.68, p_FDR < 0.001) and sleep disturbance (ρ = 0.57, p_FDR = 0.003), whereas cognition and affective symptoms were not significantly associated after correction. The motor-focused regression model showed slightly higher explanatory power (R² = 0.80).

**Conclusions:**

In advanced PD, caregiver burden is driven by motor-functional dependence and loss of autonomy, with sleep disturbance and perceived mobility-related impairment further contributing. These findings underscore the role of motor-functional disability in shaping caregiver strain in late-stage disease, highlighting the need for integrated, caregiver-centered management strategies.

## 1 Introduction

Parkinson’s disease (PD) is a progressive neurodegenerative disorder affecting approximately 1% of the population over 60 years, increasing with advancing age (Bloem et al., 2021). It is characterized by a combination of motor and non-motor symptoms that progressively compromise functional autonomy and quality of life (QoL) (Buysse et al., 1989). Bradykinesia, resting tremor, rigidity, and postural instability represent the core motor manifestations of PD (Carter et al., 2008). As the disease progresses, additional clinical features may emerge, including axial impairment, cognitive decline, neuropsychiatric disturbances, and sleep disorders, bulbar dysfunction, neuro-ophthalmological abnormalities, and respiratory disturbances. A key element supporting the differentiation of PD from other parkinsonian syndromes is the typically robust responsiveness of motor symptoms to dopaminergic therapy (Chaudhuri and Schapira, 2009). As disability increases over time, many patients progressively lose independence and become reliant on caregivers for both basic and instrumental activities of daily living (ADL) (Coelho and Ferreira, 2012, Crouse et al., 2016). The growing functional dependence associated with late-stage PD has profound consequences not only for patients but also for their informal caregivers. Caregiver burden is a multidimensional construct encompassing physical, emotional, social, and financial strain resulting from prolonged caregiving responsibilities (Davey et al., 2004). In PD, caregiver burden has been shown to be influenced by multiple factors, including motor severity, neuropsychiatric symptoms, cognitive impairment, sleep disturbances, and reduced patient autonomy (Emre, 2007). Among motor factors, postural instability and gait impairment are particularly relevant in advanced PD, as they increase fall risk, dependency, and supervision demands (Folstein et al., 1975). Functional disability in ADL and instrumental activities of daily living (IADL) has consistently been associated with increased caregiver strain (Gjerstad et al., 2018). Non-motor symptoms further contribute to disease burden. Sleep dysfunction, affecting up to 90% of individuals with PD, encompasses insomnia, fragmented sleep, REM sleep behavior disorder, and excessive daytime sleepiness (Golińska et al., 2017). Importantly, sleep disruption has been associated not only with poorer patient QoL but also with increased caregiver distress, likely reflecting nighttime caregiving demands and reduced caregiver rest (Graf, 2009).

The socioeconomic impact of advanced disease stages is considerable. Informal care costs are highest in individuals at Hoehn and Yahr (H&Y) stage IV (Hamilton, 1959), whereas those at stage V are frequently institutionalized (Hamilton, 1960).

Despite the substantial burden associated with late-stage PD, this population remains relatively understudied. Recruitment into clinical research is challenging due to the severity of motor and non-motor symptoms, advanced age, multiple comorbidities, and the tendency to disengage from specialized healthcare services in the later stages of the disease (Emre, 2007). To date, only a limited number of studies have specifically examined caregiver burden in advanced PD or atypical parkinsonism, as well as caregiver burden and satisfaction with available support (Hiseman and Fackrell, 2017, Hoehn and Yahr, 1967, Jenkinson et al., 1997). However, available evidence remains limited by relatively small samples, heterogeneous clinical assessments, and inconsistent findings regarding the relative contribution of motor symptoms, non-motor symptoms, and caregiving-related factors to caregiver burden. This uncertainty is clinically relevant because the determinants of burden may shift across the disease trajectory, with motor-functional dependence potentially becoming more prominent as disability progresses. The present study therefore aimed to examine the relative contribution of motor and non-motor symptoms to caregiver burden in individuals with late-stage PD. Specifically, we investigated whether motor-functional impairment exerts a greater impact on caregiver burden than non-motor domains, including sleep disturbance, mood symptoms, cognitive status, and health-related QoL. We hypothesized that, in advanced disease stages characterized by marked disability and dependence, motor symptoms would account for a larger proportion of variance in caregiver burden. Clarifying these determinants may inform targeted interventions to improve caregiver well-being and optimize patient care in advanced PD.

## 2 Methods

### 2.1 Study participants and design

This study included thirty people with PD (71.7 ± 6.76 years; 9 females) diagnosed according to the Movement Disorder Society (MDS) clinical criteria (Kalampokini et al., 2022). Participants were recruited from the Movement Disorders outpatient clinic and/or the Functional Rehabilitation of the IRCCS Centro Neurolesi “Bonino-Pulejo” in Messina, Italy, between February 2024 and January 2025.

Inclusion criteria consisted of a diagnosis of late-stage PD, defined as disease duration ≥ 10 years and advanced motor stage (H&Y stage IV). Additional inclusion criteria were age between 55 and 90 years and a minimum of 5 years of formal education (either primary or secondary school level). Subjects with potentially reversible forms of parkinsonism, such as normal pressure hydrocephalus or drug-induced parkinsonism, were excluded from the study.

Eligible patients were assessed individually in a single comprehensive session by a multidisciplinary team consisting of a clinical psychologist, physiotherapist and a neurologist specialized in movement disorders. The assessment was conducted in a defined dopaminergic “OFF” state. Demographic and clinical information was collected, including age, sex and years of education.

Thirty caregivers (63.8 ± 13.84 years; 18 females) participated in the study. Eligibility criteria for caregivers included being at least 18 years of age, having no significant cognitive impairments, and being free from serious medical conditions. All caregivers were cohabiting with the patients. Regarding their relationship to the patients, most caregivers were spouses or partners (n = 22; 14 wives and 8 husbands), followed by adult children (n = 7; 3 daughters and 4 sons) and one sibling (sister).

The study was approved by the Ethics Committee of the IRCCS Centro Neurolesi “Bonino-Pulejo” (approval No. E6/2024) on February 6, 2024 and was conducted in accordance with the principles of the Declaration of Helsinki. All participants provided written informed consent prior to participation, and confidentiality of personal data was ensured.

### 2.2 Clinical assessment

#### 2.2.1 Procedure and clinical evaluation

All participants with PD underwent a standardized neurological examination that included disease staging according to the H&Y scale. In addition, a comprehensive battery of neuropsychological, motor, and functional assessments was administered in order to evaluate cognitive performance, mood symptoms, sleep quality, health-related QoL, and functional autonomy in daily activities.

Global cognitive functioning was assessed using both the Mini-Mental State Examination (MMSE) (Katz, 1983) and the Montreal Cognitive Assessment (MoCA) (Kegelmeyer et al., 2007). The MMSE is a widely used screening instrument that evaluates orientation, memory, attention, language, and visuospatial abilities, with total scores ranging from 0 to 30 and scores above 24 generally considered indicative of normal cognitive functioning. The MoCA is a brief 30-point screening tool designed to assess multiple cognitive domains, including executive functions, attention, memory, and language; a total score of 26 or higher is typically considered within the normal range.

Mood and psychological symptoms were evaluated using clinician-rated instruments. Depressive symptoms were assessed with the Hamilton Rating Scale for Depression (HAM-D) (Klietz et al., 2020), which yields scores ranging from 0 to 52, with scores ≥ 17 indicating clinically significant depression. Anxiety symptoms were measured using the Hamilton Rating Scale for Anxiety (HAM-A) (Lana et al., 2007), a 56-point scale in which scores ≥ 18 reflect clinically relevant anxiety.

Sleep quality was assessed using the Pittsburgh Sleep Quality Index (PSQI) (Macchi et al., 2020), a self-report questionnaire that evaluates sleep quality and disturbances over the previous month. The PSQI comprises seven components, including sleep latency, duration, and disturbances, and provides a global score, with higher values indicating poorer sleep quality.

Health-related quality of life was measured using the Parkinson’s Disease Questionnaire (PDQ-39) (Mahoney and Barthel, 1965), a disease-specific self-administered instrument assessing eight domains, including mobility, activities of daily living, emotional well-being, and social support. Total scores range from 0 to 156, with higher scores reflecting greater impairment; interpretation is based on overall and domain-specific distributions rather than a fixed clinical cut-off.

Functional autonomy was evaluated using both the Activities of Daily Living (ADL) scale (Martínez-Martín et al., 2007) and the Instrumental Activities of Daily Living (IADL) scale according to Lawton and Brody criteria (Martinez-Martin et al., 2023, McLaughlin et al., 2011). The ADL scale assesses independence in six basic self-care activities such as bathing, dressing, toileting, transferring, continence, and feeding, yielding a total score from 0 (complete dependence) to 6 (full independence), with lower scores indicating greater disability. The IADL scale extends this evaluation to more complex daily tasks necessary for independent community living, including telephone use, shopping, food preparation, housekeeping, laundry, transportation, medication management, and financial handling. Total IADL scores range from 0 to 8, with lower scores reflecting greater functional limitation.

ADL/IADL scales were included to capture the patient’s overall level of independence in basic and instrumental daily activities, whereas the Barthel Index was used as a complementary measure providing a more graded assessment of basic functional performance, including mobility-related components such as transfers, ambulation, and stair climbing.

Motor performance was assessed using standardized clinical measures of balance, gait, and functional mobility. Balance and gait were assessed using the Tinetti Performance-Oriented Mobility Assessment (POMA), whereas the Barthel Index was used to provide a graded assessment of basic functional performance and mobility-related independence (Mosley et al., 2017, Novak and Guest, 1989). During the Tinetti POMA, participants underwent a standardized set of balance and gait tasks that cover functional transitions, static and dynamic balance and walking. For each item, scores ranged from 0 to 2 points, with higher scores indicating better performance. The balance subscale includes 9 items (0–16 points) and the gait subscale includes 7 items (0–12 points), for a total score of 0–28. The Barthel Index evaluates basic ADL across 10 domains: feeding, bathing, grooming, dressing, bowel control, bladder control, toilet use, transfers (bed–chair), mobility on level surfaces, and stair climbing. Items are weighted (e.g., scored in steps of 0–5, 0–10, or 0–15 depending on the task), yielding a total score ranging from 0 to 100, with higher scores indicating greater independence in ADL. Both instruments have shown acceptable-to-good validity and reliability in neurological populations, including people with PD (Olson et al., 2025, Opara et al., 2017, Perez et al., 2022).

Caregiver burden was evaluated using the Caregiver Burden Inventory (CBI) (Pinquart and Sorensen, 2007), a multidimensional self-report questionnaire originally developed for caregivers of individuals with dementia. The CBI consists of 24 items rated on a 5-point Likert scale and captures five dimensions of burden: time-dependence, developmental, physical, social, and emotional burden. Higher total scores indicate greater overall caregiver burden. Although not specifically validated in PD populations, the CBI has demonstrated sensitivity to changes following psychosocial interventions in caregivers of individuals with PD (Hamilton, 1960) supporting its applicability in this context. Its multidimensional structure allows for a nuanced assessment of the diverse stressors experienced by caregivers, which is particularly relevant in advanced stages of PD, where care demands often encompass both substantial physical assistance and emotional strain.

### 2.3 Statistical analysis

Normality of continuous variables was assessed using the Shapiro–Wilk test. Given the modest sample size and frequent departures from normality, associations between caregiver burden (CBI total score and subscales) and clinical measures were primarily quantified using Spearman’s rank correlation coefficients, while, Pearson correlations were computed only when both variables were approximately normally distributed. Multiple testing was addressed by controlling the false discovery rate (FDR) separately within each burden outcome (CBI total and each CBI subscale). To explore independent predictors of caregiver burden, multivariable linear regression models were fitted with the CBI total score as the dependent variable. A prespecified main model included motor/functional, non-motor, sleep and QoL variables, along with patient and caregiver demographic covariates; three additional domain-specific models were estimated focusing on sleep, QoL and motor/functional indices, respectively. To reduce multicollinearity and improve model stability in relation to sample size, predictors were pruned using variance inflation factor (VIF) criteria (threshold = 5), retaining variables below a predefined collinearity threshold. Regression coefficients were estimated using heteroscedasticity-consistent (HC-type) robust standard errors. Model performance was summarized using adjusted R² and Akaike Information Criterion (AIC). Assumptions were evaluated via residual diagnostics, including Shapiro–Wilk tests for residual normality, Breusch–Pagan tests for heteroscedasticity, and inspection of Cook’s distance, leverage and studentized residuals to identify influential observations. All tests were two-tailed, and statistical significance was set at p < 0.05 after correction where applicable. Analyses were conducted in R (version 4.4.2).

## 3 Results

### 3.1 Correlation analyses

**Fig. 1:**
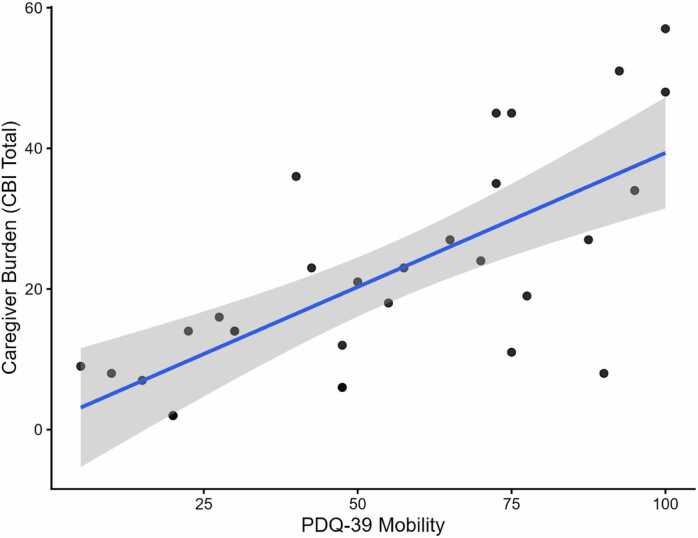
Association between caregiver burden and mobility-related quality of life. Scatterplot showing the relationship between caregiver burden (CBI total score) and PDQ-39 Mobility. The solid line represents the fitted linear regression line and the shaded area indicates the 95% confidence interval.

Analyses were performed in 30 patient–caregiver pairs. Correlations between caregiver burden and clinical variables were primarily evaluated using Spearman’s rank coefficients with false discovery rate correction applied within each burden outcome. For the CBI total score, the strongest association was observed with caregiving duration (ρ = 0.81, p < 0.001, p_FDR < 0.001), indicating higher burden with longer time spent providing care. Greater burden was also linked to reduced patient autonomy, with significant inverse correlations with IADL (ρ = −0.76, p < 0.001, p_FDR < 0.001) and ADL (ρ = −0.63, p < 0.001, p_FDR < 0.001). Motor impairment showed consistent associations with caregiver burden, as reflected by significant negative correlations with the Tinetti total score (ρ = −0.67, p < 0.001, p_FDR < 0.001), Tinetti balance (ρ = −0.65, p < 0.001, p_FDR < 0.001), Tinetti gait (ρ = −0.62, p < 0.001, p_FDR = 0.001), and the Barthel Index (ρ = −0.47, p = 0.009, p_FDR = 0.02). Among non-motor domains, burden increased with worse health-related QoL, particularly PDQ-39 Mobility (ρ = 0.68, p < 0.001, p_FDR < 0.001) (Fig. 1) and PDQ-39 ADL (ρ = 0.55, p = 0.002, p_FDR = 0.005), and with sleep disturbance, including the global PSQI score (ρ = 0.57, p < 0.001, p_FDR = 0.003) and the sleep disturbances subscale (ρ = 0.62, p < 0.001, p_FDR = 0.001). After correction, no significant associations were observed for affective symptoms (HAM-D/HAM-A) or global cognitive measures (MoCA/MMSE).

### 3.2 Burden subscales

Patterns for the CBI subscales were broadly concordant with the total score. The evolutionary burden subscale was strongly associated with caregiving duration (ρ = 0.73, p < 0.001, p_FDR < 0.001) and showed significant links with disability and gait/balance performance, including IADL (ρ = −0.68, p < 0.001, p_FDR < 0.001) and Tinetti measures (|ρ| ≈ 0.54–0.71; all p_FDR < 0.01). The physical burden subscale showed more modest associations that did not consistently survive after correction, suggesting a weaker and potentially more variable relationship with the examined clinical domains in this sample.

### 3.3 Multivariable regression analyses

**Fig. 2:**
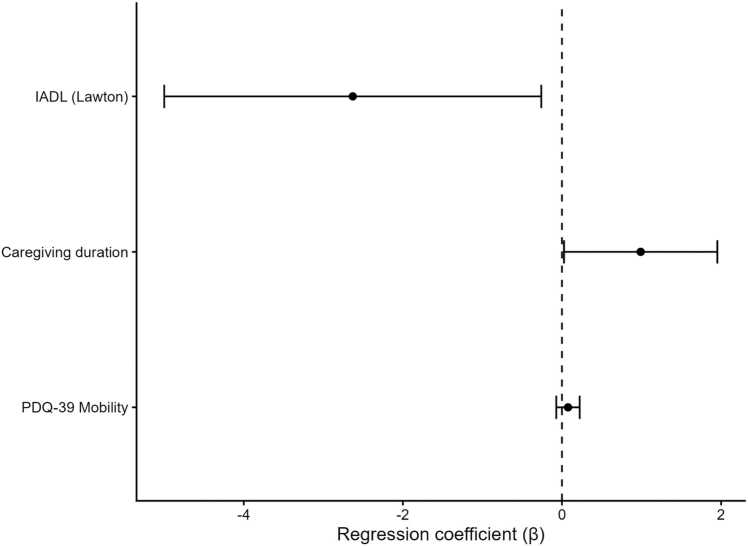
Main multivariable model for caregiver burden. Forest plot of regression coefficients (β) from the main multivariable linear model with 95% confidence intervals, showing the direction and magnitude of associations between selected predictors and CBI total score. Positive coefficients indicate higher burden with increasing predictor values, whereas negative coefficients indicate lower burden.

Multivariable linear regression models were fitted to estimate independent contributors to caregiver burden across motor, sleep and QoL domains, while controlling for multicollinearity through VIF-based pruning and estimating coefficients with robust (HC-type) standard errors. Model fit and diagnostic indices for the main and domain-specific regression models are reported in Table 1. Overall model performance was high, with explained variance ranging from R² = 0.74–0.80 and adjusted R² = 0.71–0.77 across the tested models. The motor model showed the best fit (R² = 0.80; adj. R² = 0.77) and the most favorable information criteria (AIC = 210.61), followed by the pruned main model (R² = 0.786; adj. R² = 0.76; AIC = 212.14). The sleep-only model also performed well (R² = 0.777; adj. R² = 0.751; AIC = 213.46), whereas the QoL-only model showed the lowest fit (R² = 0.74; adj. R² = 0.71; AIC = 217.60). After collinearity-driven pruning, the main model retained a parsimonious set of predictors (including caregiving duration, IADL, and PDQ-39 Mobility), with effect directions consistent with the correlation analyses, however, several coefficients did not reach conventional statistical significance. Model diagnostics supported the adequacy of assumptions, residual normality was acceptable across models (Shapiro–Wilk p = 0.22–0.65) and there was no evidence of heteroscedasticity (Breusch–Pagan p = 0.14–0.53). Adjusted estimates from the main multivariable model are reported in Fig. 2.

**Table 1:** Multivariable linear regression model performance and diagnostics for caregiver burden (CBI total).

**Model**	**R²**	**Adjusted R²**	**AIC**	**Breusch–Pagan p**
Motor_only	0.80	0.77	210.61	0.53
Main model (Motor-sleep-QoL-covariate)	0.79	0.76	212.14	0.17
Sleep_only	0.77	0.75	213.46	0.52
QoL_only	0.74	0.71	217.60	0.14

Legend: AIC = Akaike Information Criterion.

## 4 Discussion

The present study investigated the clinical determinants of caregiver burden in a cohort of individuals with late-stage PD, focusing on motor disability, functional autonomy, sleep disturbances, and health- related QoL. Our findings indicate that caregiver burden is strongly associated with caregiving duration, reduced patient autonomy in both ADL and IADL, impaired gait and balance performance, sleep disturbance, and mobility-related QoL. In contrast, global cognitive measures and affective symptoms were not significantly associated with caregiver burden. Importantly, these results contribute to the ongoing debate regarding the relative contribution of motor and non-motor symptoms, suggesting that in advanced disease stages, motor-functional impairment represents one of the most influential contributors to caregiver strain.

A central finding of this study is the strong association between caregiver burden and reduced functional autonomy, particularly IADL. Functional disability has consistently been identified as a primary determinant of caregiver strain in PD (Santangelo et al., 2015). In advanced stages of the disease, dependence in instrumental tasks such as medication management, transportation, and financial handling substantially increases the time commitment and responsibility placed on caregivers (Santos-García and de la Fuente-Fernández, 2015). Beyond the physical effort involved, these tasks often entail continuous supervision, decision-making responsibilities, and coordination of care, thereby amplifying perceived burden. The robust inverse correlations observed with both IADL and ADL scores reinforce the notion that loss of autonomy represents a key driver of burden in late-stage PD. These results are aligned with stress-process models of caregiving, which conceptualize objective care demands as proximal determinants of caregiver strain (Shin and Habermann, 2020).

Motor impairment, particularly in the domains of gait and balance, also emerged as a significant contributor to caregiver burden. Postural instability and gait dysfunction represent hallmark features of advanced PD and are strongly associated with falls, injury risk, and increased need for supervision (Smith et al., 1997). The consistent associations observed with motor scales suggest that mobility-related impairment directly translates into increased caregiver demands. Notably, the motor-focused regression model showed slightly higher explanatory power than other domain-specific models, supporting an important role of motor-functional disability in shaping caregiver experiences in this population. These findings highlight the clinical relevance of interventions aimed at preserving mobility and reducing fall risk, which may have indirect benefits for caregiver well-being.

However, the literature reports heterogeneous findings regarding the relative contribution of motor symptoms to caregiver burden. Several studies have identified motor severity and functional disability as major determinants of caregiver strain in PD (Hamilton, 1960, Spector et al., 1987, Sprajcer et al., 2022), supporting the view that increasing motor dependence substantially amplifies caregiving demands. In contrast, other investigations have suggested that non-motor symptoms, particularly neuropsychiatric manifestations such as depression, apathy, psychosis, and cognitive impairment, may exert a stronger impact on caregiver burden than motor impairment per se (Taghizadeh et al., 2020, Tan et al., 2019). Such discrepancies may be attributable to differences in disease stage, as non-motor symptoms appear to play a more prominent role in earlier or cognitively complicated phases, whereas motor-functional disability may become the predominant driver of burden once physical dependence intensifies in advanced PD.

Mobility-related QoL, as measured by the PDQ-39 Mobility subscale, showed one of the strongest positive associations with caregiver burden. The PDQ-39 captures the patient’s subjective perception of disease impact, which may reflect daily caregiving challenges more directly than objective motor scales alone (Tessitore et al., 2018). The persistence of mobility-related QoL in the pruned multivariable model suggests that perceived disability and lived experience of motor impairment may influence caregiver stress beyond performance-based measures. This finding highlights the importance of integrating both objective clinical assessments and patient-reported outcomes when evaluating determinants of caregiver burden.

Caregiving duration showed the strongest correlation with total burden, highlighting the cumulative psychological and physical toll of prolonged caregiving. Chronic exposure to sustained care demands may progressively deplete coping resources and resilience, leading to heightened perceived burden over time (Tinetti, 1986). In advanced PD, the increasing need for physically demanding tasks, such as feeding, lifting, and transferring the patient, further amplifies the strain associated with long-term caregiving. Moreover, the advanced age of many caregivers, the presence of comorbid medical conditions, and the cumulative impact of psychological distress may contribute to declining physical health and increased vulnerability over time (Torny et al., 2018, Tysnes and Storstein, 2017). These findings underscore the importance of early caregiver support interventions and continuous monitoring throughout the disease trajectory.

Sleep disturbance was another domain significantly associated with caregiver burden. Higher global sleep disturbance scores were correlated with increased burden, suggesting that nocturnal symptoms may represent an independent stressor in advanced PD. Nighttime motor symptoms and behavioral disturbances can disrupt not only patient sleep but also that of caregivers, leading to cumulative fatigue and emotional strain (Váradi, 2020). Literature indicates that sleep disturbances among spouses of individuals with PD may affect up to 27% of male caregivers and 48% of female caregivers, rates comparable to those observed in patients with PD (Zarit et al., 1986). One plausible explanation is the frequent need for caregivers to remain awake during the night to assist patients experiencing disrupted sleep. The relatively strong performance of the sleep-only regression model further supports the relevance of nocturnal symptom management as a potential target for reducing caregiver burden.

From a clinical perspective, these findings indicate that caregiver strain in late-stage PD is largely shaped by progressive physical dependence, sleep disruption, and sustained caregiving demands. Motor-functional impairment emerged as the most influential domain in multivariable analyses, underscoring the central role of mobility limitations and loss of autonomy in determining caregiver burden at advanced stages of the disease. These results highlight the importance of integrated care strategies that extend beyond symptom management to include systematic assessment of caregiver well-being, structured psychosocial support, and targeted interventions aimed at preserving patient mobility and optimizing sleep quality. Taken together, these findings suggest that in advanced PD, the balance shifts toward motor-functional dependence as the central determinant of caregiver strain, emphasizing the need for multidisciplinary models of care that address both patient disability and caregiver resilience throughout the disease trajectory.

## 5 Limitations

Several limitations should be acknowledged. The modest sample size limits generalizability and statistical power. The cross-sectional design precludes causal inference and does not allow conclusions regarding the directionality of the observed associations. Although the CBI is a multidimensional instrument, it has not been specifically validated in PD populations, despite prior evidence supporting its sensitivity in this context (Hamilton, 1960). Motor impairment was assessed using standardized functional measures, which may not fully capture Parkinson-specific motor features particularly relevant to caregiving demands, such as freezing of gait, turning and transitional movements or mobility under dual-task conditions. Furthermore, the study did not include specific measures assessing caregivers’ emotional well-being, psychological distress, or coping strategies. The absence of these variables limits our ability to explore the potential moderating or mediating role of individual psychological factors in shaping perceived burden. Future longitudinal studies incorporating comprehensive assessments of caregiver emotional functioning, coping mechanisms, and Parkinson-specific motor measures are warranted to clarify temporal relationships, identify modifiable predictors of burden progression and better reflect real-world caregiving demands.

## 6 Conclusion

In advanced PD, caregiver burden is largely driven by increasing physical dependence and prolonged caregiving exposure, with sleep disruption representing an additional stressor. In this context, motor-functional impairment assumes a dominant role in determining caregiver strain. These results underscore the importance of integrated, caregiver-centered care models in late-stage disease.

## CRediT authorship contribution statement

**Roberta Lombardo:** Writing – review & editing, Methodology. **Gabriele Triolo:** Writing – review & editing, Methodology. **Lilla Bonanno:** Writing – review & editing, Formal analysis, Data curation. **Daniela Ivaldi:** Writing – review & editing, Methodology. **Viviana Lo Buono:** Visualization, Validation, Supervision. **Silvia Marino:** Visualization, Validation, Supervision. **Laura Culicetto:** Writing – original draft, Methodology, Conceptualization. **Giuseppe Di Lorenzo:** Visualization, Validation. **Fabio Mauro Giambò:** Writing – review & editing, Data curation. **Angelo Quartarone:** Visualization, Validation. **Chiara Sorbera:** Visualization, Validation.

## Institutional review board statement

The study was conducted in accordance with the Declaration of Helsinki and approved by the Ethics Committee of the IRCCS Centro Neurolesi “Bonino-Pulejo” (Approval No. E6/2024, approved on 6 February 2024, Clinical trial registration: NCT06341829).

## Informed consent statement

This study was conducted according to the Declaration of Helsinki. Written informed consent was obtained from the individuals for the publication of any potentially identifiable data included in this article.

## Funding statement

This study was supported by Current Research Funds 2026, RRC-2026–23688274 10.13039/501100003196Ministry of Health, Italy.

## Declaration of Competing Interest

The authors declared no potential conflicts of interest with respect to the research, authorship, and/or publication of this article.

## Data Availability

The data associated with the paper are not publicly available but are available from the corresponding author on reasonable request.
